# Pump–probe reciprocal-space mapping using energy-resolved XFEL pink beam pulses

**DOI:** 10.1107/S1600577525000463

**Published:** 2025-02-12

**Authors:** Ouyoung Kwon, Sungsoo Ha, Do Young Noh, Hyonchol Kang, Mazhar Iqbal, Muhammad Ijaz Anwar, Sunam Kim

**Affiliations:** a Department of Physics and Photon Science Gwangju Institute of Science and Technology Gwangju61005 Republic of Korea; b Department of Materials Science and Engineering Chosun University Gwangju61452 Republic of Korea; c National Institute of Lasers & Optronics (NILOP), Islamabad22869, Pakistan; d PAL-XFEL Division, Pohang Accelerator Laboratory, Pohang37673, Republic of Korea; RIKEN SPring-8 Center, Japan

**Keywords:** 3D reciprocal-space mapping, pink SASE XFELs, energy-resolved pulses, pump–probes

## Abstract

We present a 3D reciprocal-space mapping (RSM) method using a pink self-amplified spontaneous emission (SASE) X-ray free-electron laser beam. The energy of each specific pulse in a SASE beam can be determined using the diffraction pattern of a specimen excited by pumping itself as a spectroscopic reference. A thin slab of RSM, whose thickness corresponds to the energy bandwidth of the pink beam, is successfully reconstructed using the proposed method. By rocking a sample in a few steps, we obtained a 3D RSM covering both the diffuse scattering and the Bragg rod in NiO thin films during a pump–probe X-ray diffraction measurement.

## Abstract 2

Pump–probe 3D X-ray reciprocal-space mapping (RSM) is devised using energy-resolved pulses in self-amplified spontaneous emission X-ray free-electron laser beams. Extended 3D RSMs covering both the diffuse scattering and the Bragg rod in an NiO thin film were obtained.

## 1. Introduction

Recently, X-ray free-electron lasers (XFELs) have opened numerous avenues for probing ultrafast dynamic processes in nature. With unprecedented brightness, coherence and time structure, XFEL sources have revealed various atomic-scale phenomena (McNeil & Thompson, 2010 ▸; Emma *et al.*, 2010 ▸; Orville, 2020 ▸). In particular, the ultra-short time structure of high-energy X-ray pulses from XFELs has provided a great advantage in dissecting the timescale of atomic dynamics of materials down to femtoseconds (Spence, 2017 ▸; Husband *et al.*, 2021 ▸). Combined with X-ray diffraction techniques, XFEL sources have led to valuable discoveries (Inoue *et al.*, 2016 ▸; Tanaka *et al.*, 2013 ▸; Shayduk *et al.*, 2016 ▸; Assefa *et al.*, 2020 ▸).

Many experiments at XFELs utilizing a self-amplified spontaneous emission (SASE) beam have been conducted with a monochromated beam rather than the raw SASE beam, often referred to as a ‘pink beam’ due to the finite energy bandwidth of ∼0.3% (Min *et al.*, 2019 ▸). The X-ray energy of the SASE XFEL beam fluctuates pulse-by-pulse causing the probing point to wander around reciprocal space, blurring probed features in diffraction experiments (Tono *et al.*, 2013 ▸; Karvinen *et al.*, 2012 ▸). For these reasons, high-resolution monochromators, which offer energy bandwidths of ∼10^−4^, have often been employed when using SASE XFEL beams in many experiments (Tschentscher *et al.*, 2017 ▸; Ko *et al.*, 2017 ▸; Yabashi *et al.*, 2015 ▸). Although a monochromated beam loses a significant amount of photon flux, by a factor of about ∼30 (Osaka *et al.*, 2019 ▸), employing a monochromatic beam has been considered a necessary trade-off for more accurate measurements.

Self-seeded XFELs that provide longitudinally coherent monochromatic X-ray beams with superior spectral brightness have been successfully demonstrated (Liu *et al.*, 2019 ▸; Min *et al.*, 2019 ▸). Furthermore, novel techniques that expand the notion of seeding, such as external seeding or utilizing optical cavity, have been actively discussed in recent years (Yu *et al.*, 2000 ▸; Xiang *et al.*, 2010 ▸; Kim *et al.*, 2008 ▸; Yang *et al.*, 2023 ▸; Rauer *et al.*, 2023 ▸). Although seeded XFELs have enlarged the application areas for monochromatic beams, their availability is still scarce due to limited operating resources and related technical difficulties. The pink XFEL beam still remains attractive due to its unmatched photon flux for photon-hungry ultrafast measurements.

In this paper, we present a method that employs SASE beams in diffraction experiments, wherein we unravel the energy of individual XFEL pulses using the diffraction pattern from a specimen itself as a spectroscopic reference. This method not only allows for full utilization of the high photon flux of SASE XFEL beams but also enables probing of 3D reciprocal space, albeit with a limited range. We applied this methodology to a pump–probe X-ray diffraction study of thin NiO films and successfully mapped an extended reciprocal-space volume using the pink XFEL beam.

In the following sections, we elaborate on the procedures used to determine the energy of the pulses in a pink XFEL beam and reconstruct a 3D reciprocal-space map (RSM). First, the experimental setup is introduced along with the pulse energy measurement scheme. In the *Results and discussion*[Sec sec3] section, we present a 3D RSM obtained using the proposed method, which includes both the diffuse scattering and the Bragg rod from the thin NiO film.

## 2. Experimental procedure

### 2.1. Individual pulse energy determination scheme

The mean energy 〈*E*〉 of a single SASE XFEL pulse, with its internal energy structure, can be determined by measuring the Bragg angle θ of a Bragg peak of a crystalline specimen using Bragg’s law, 〈*E*〉 = 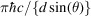, where *d* is the lattice spacing of the specimen. Since the energy of a specific pulse is unknown in advance, and the measurement with that specific pulse cannot be repeated, a specimen with a finite mosaic spread is preferable as it is difficult to orient a single-crystal specimen exactly at the Bragg angle.

**Figure 1:**
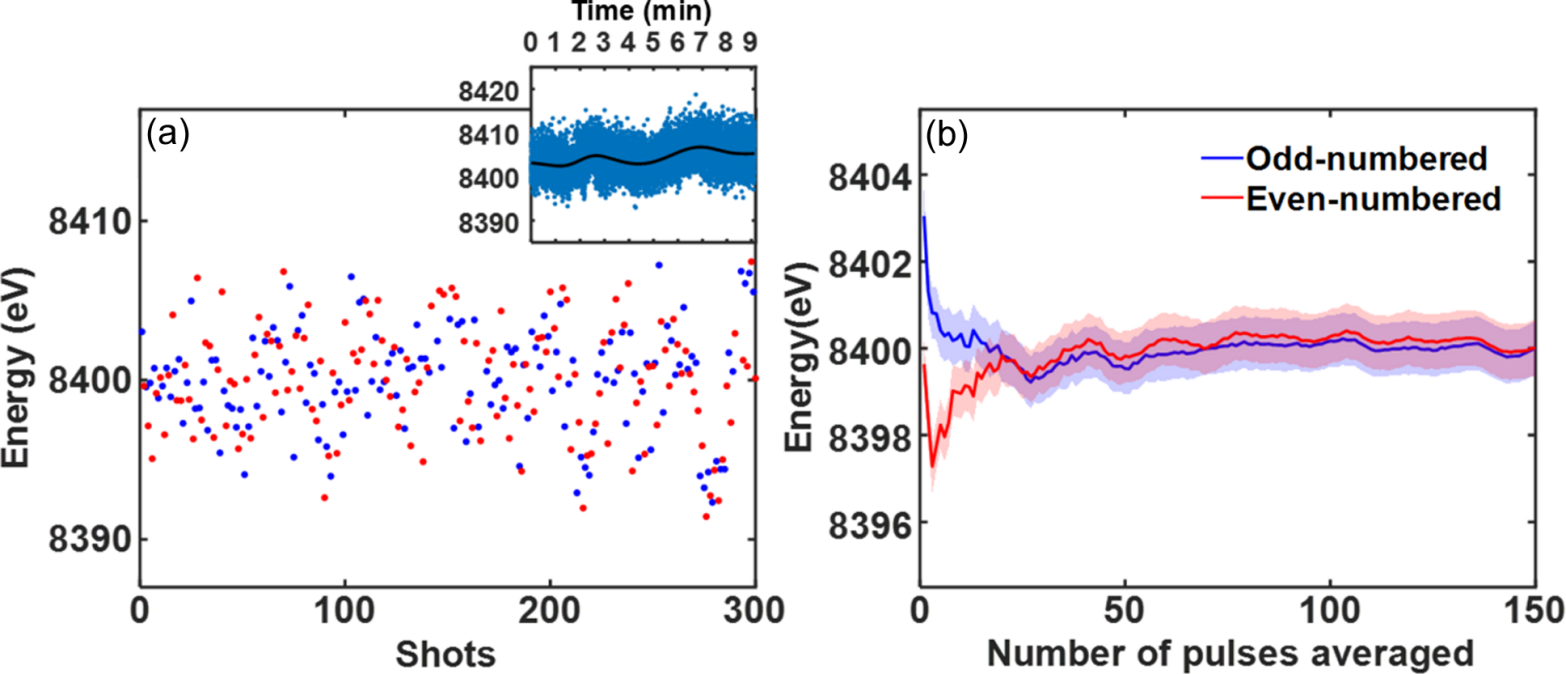
(*a*) Energy of individual pulses in an XFEL pink beam obtained from the peak position of the NiO (111) Bragg reflection. Blue and red dots indicate odd- and even-numbered pulses, respectively. The pulse energies in a long timescale are shown in the inset. The local averaged values shown as the black-solid line indicate the long-term drift. (*b*) Local average energy of the odd- and even-numbered pulses plotted as a function of the number of pulses included in averaging. The 95% confidence interval is indicated by the shaded areas.

Fig. 1 ▸(*a*) shows the pulse-to-pulse energy of the XFEL pulses provided by the PAL-XFEL operating at 30 Hz determined by the Bragg angle of the (111) reflection from a thin NiO film with a mosaic spread of about 0.5°. The sample was fabricated by electron-beam deposition on a sapphire substrate. A two-dimensional CCD detector (Kameshima *et al.*, 2014 ▸) with 512 × 1024 pixels with a size of 50 µm × 50 µm was placed 50 cm downstream of the specimen to obtain the diffraction profile. The energy of each pulse fluctuates around a local averaged value that drifts up and down in a relatively long timescale compared with the pulse interval. The local energy jitter lies within approximately 10 eV, while the long-term energy drift was about 30 eV.

The local average energies of the odd- and even-numbered pulses are shown in Fig. 1 ▸(*b*) as a function of the number of pulses included in averaging. This demonstrates that the average energies of the odd- and even-numbered pulses are essentially the same within about 1 eV when more than 100 pulses are included in the averaging. In a typical pump–probe XFEL experiment, one delivers a pump pulse to a specimen for excitation, and probes its dynamic behavior using a probing X-ray pulse after a controlled time delay. To normalize the state of a specimen, unpumped (cold) and pumped (hot) states of the specimen are alternately probed in actual experiments, assuming the specimen has fully recovered from the previous hot state. Based on Fig. 1 ▸(*b*), one may assume that the local average energy of hot pulses is the same as that of cold pulses.

The energy of each individual pulse used for a cold state can simply be obtained from the Bragg peak position since its lattice spacing is presumed to be known as in Fig. 1 ▸(*a*). However, it is difficult to assign the energy of individual hot pulses, as the lattice spacing in a hot state changes dynamically due to the influence of a pumping source. However, we note that the local average energy of hot pluses, averaged over about 100 pulses used for a given time delay, 〈*E*〉_local_av_, is the same as the average of the cold pluses intervening them as demonstrated in Fig. 1 ▸(*b*). It is therefore possible to determine the dynamic lattice spacing at a given time delay, *d*_*h*_, from the Bragg peak profile accumulated by a number of repeated pump–probe measurements using the local averaged energy value obtained from cold pulses. Then, the energy of each specific pulse used to probe the hot state can consequently be assigned from the Bragg angle θ_pulse_ of the peak produced by itself using
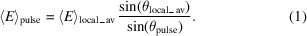
The resolution of the mean energy of an individual pulse can be estimated from the accuracy in determining the Bragg angle, θ_pulse_, and the energy profile of individual pulses. The error bars shown in Fig. 1 ▸(*b*) are the uncertainties in the mean energy of individual pulses at the 95% confidence level resulting from the fitting of the Bragg peak position, which is about 1 eV. The energy profile of single SASE pulses should be deconvolved when more detailed peak characteristics beyond peak position are desired. In this experiment, 150 pulses are used at each time delay, covering an energy range of about 20 eV. The pulses are finely distributed in energy and are thus discretely binned to represent a specific mean energy. By setting each bin as ±1 eV around a specific energy, about 15 pulses are accumulated in each bin. Therefore, the average energy profile of the accumulated pulses in a given bin is convolved to each data point, which may be termed the energy resolution. While it is ideal to incorporate a high-resolution spectrometer to measure the resolution prior to a sample study, one may approximately use a Gaussian with a width of the SASE energy spread, which is typically 5 eV (half width at half-maximum) (Kim *et al.*, 2025 ▸). This approximation should be carefully applied to scarcely binned data points near the edge of the SASE energy spread. We note that the actual lattice constant of a specimen in the cold state can be different from the known lattice spacing due to defects or remnant heat, and one needs to check the cold state to avoid potential errors in the energy determination. Pre-obtained information on the RSM of the cold state is desirable for accurate energy calibration.

### 2.2. Experimental setup

**Figure 2:**
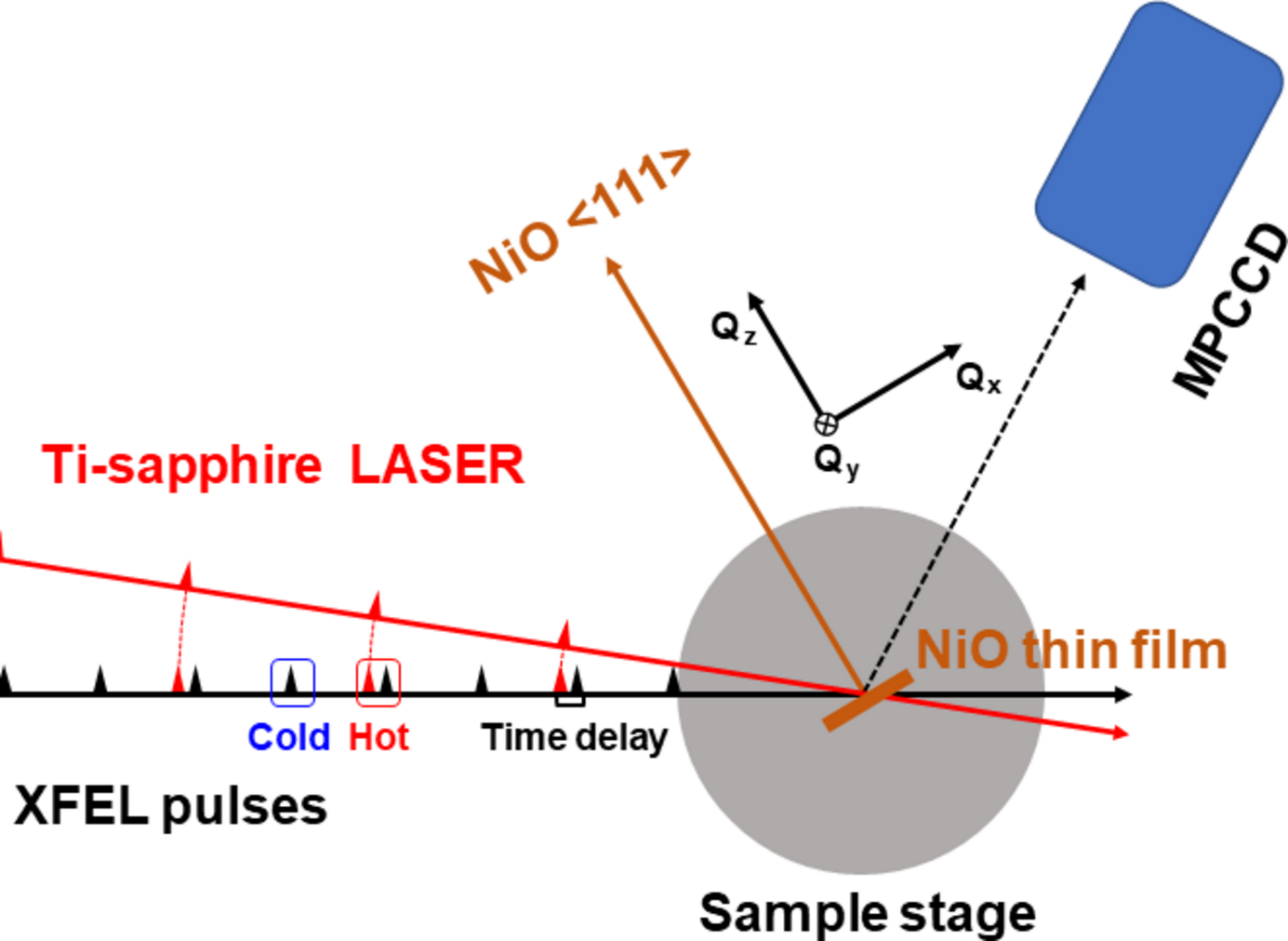
Schematic of the experimental setup used in the pump–probe measurements at PAL XFEL. Measurements were carried out as a function of the time delay between the pumping laser pulse in red and the probing hot X-ray pulses. In between hot pulses, there exists cold pulses to measure the specimen in an unperturbed state.

In order to validate the proposed scheme, we carried out a laser pump X-ray probe experiment at the PAL-XFEL using NiO thin films grown on sapphire substrates. As an excitation pumping source, a Ti-sapphire femtosecond laser was used which provided 30 fs laser pulses of 1.5 eV energy at 15 Hz. The time delay between XFEL and laser pulses was controlled in a few picoseconds range. At each time delay, X-ray pulses with laser illumination (hot shot) and pulses without laser illumination (cold shot) were delivered alternatively to the specimen; this was repeated for 150 times. The pump–probe scheme used in this experiment is illustrated in Fig. 2 ▸.

**Figure 3:**
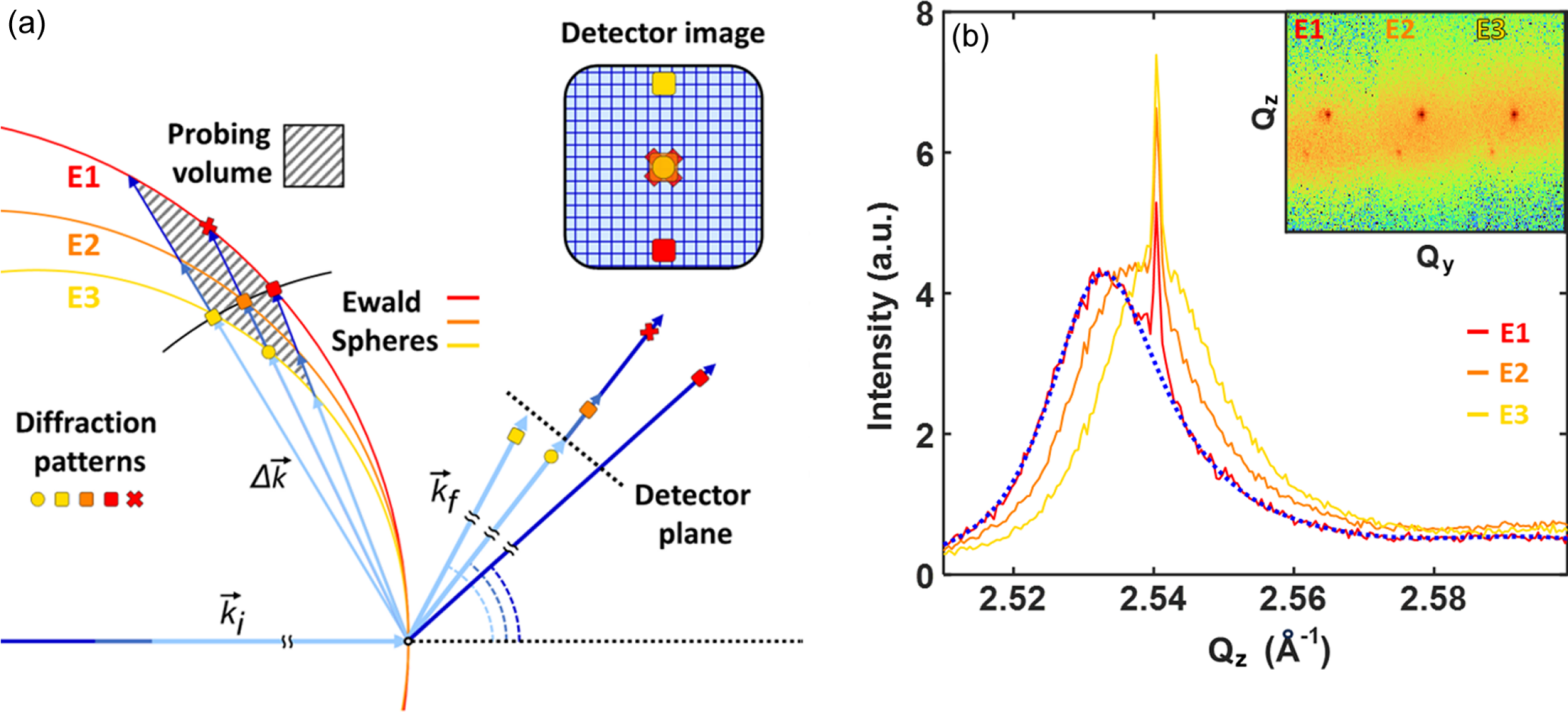
(*a*) Probed volume in reciprocal space with the Ewald spheres of three different X-ray energies. The volume probed by the momentum transfer **Q** using X-rays of a finite energy band is indicated by the hatched box. The diffracted intensity spots recorded on the detector represent the values at the corresponding positions indicated in the same colors in reciprocal space. (*b*) Diffraction profile of the NiO thin film corresponding to the scheme illustrated in (*a*) measured with three individual pulses. *Q*_*z*_ profiles of the diffraction pattern are plotted with the inset showing the captured images on the detector. The dotted blue line is the result of the fit with a fitting error in the peak position that is translated into ∼1 eV in energy.

Each detector pixel in the detector captures diffracted X-ray photons in a specific direction, and probes an individual point **Q** in reciprocal space through **Q** ≡ **k**_f_ − **k**_i_, where **k**_i(f)_ is the incident (diffracted) X-ray wavevector. As the energy of X-ray photon determines the magnitude of the wavevector, |**k**|, the collection of the diffraction intensities caused by a single X-ray pulse recorded on the pixels in a detector probes a portion of the Ewald sphere surface as illustrated in Fig. 3 ▸.

When pulses of various energies in a pink beam are employed, the probing region in reciprocal space expands to a surface shell of the Ewald sphere, if the energy of each pulse is resolved and recorded. The width of the shell is determined by the energy bandwidth of the pink XFEL beam, and it is possible to obtain a 3D RSM in a limited range as illustrated schematically in Fig. 3 ▸. The trace of **Q** probed by a pixel at a fixed diffraction angle draws a straight line, namely an equi-pixel line, across the Ewald sphere. The intensity profile along the equi-pixel line follows the energy profile of the pink beam. This is well described in the experiment data from three different energies depicted in Fig. 3 ▸(*b*), which shows the static Bragg rod with varying diffuse peak position.

## 3. Results and discussion

**Figure 4:**
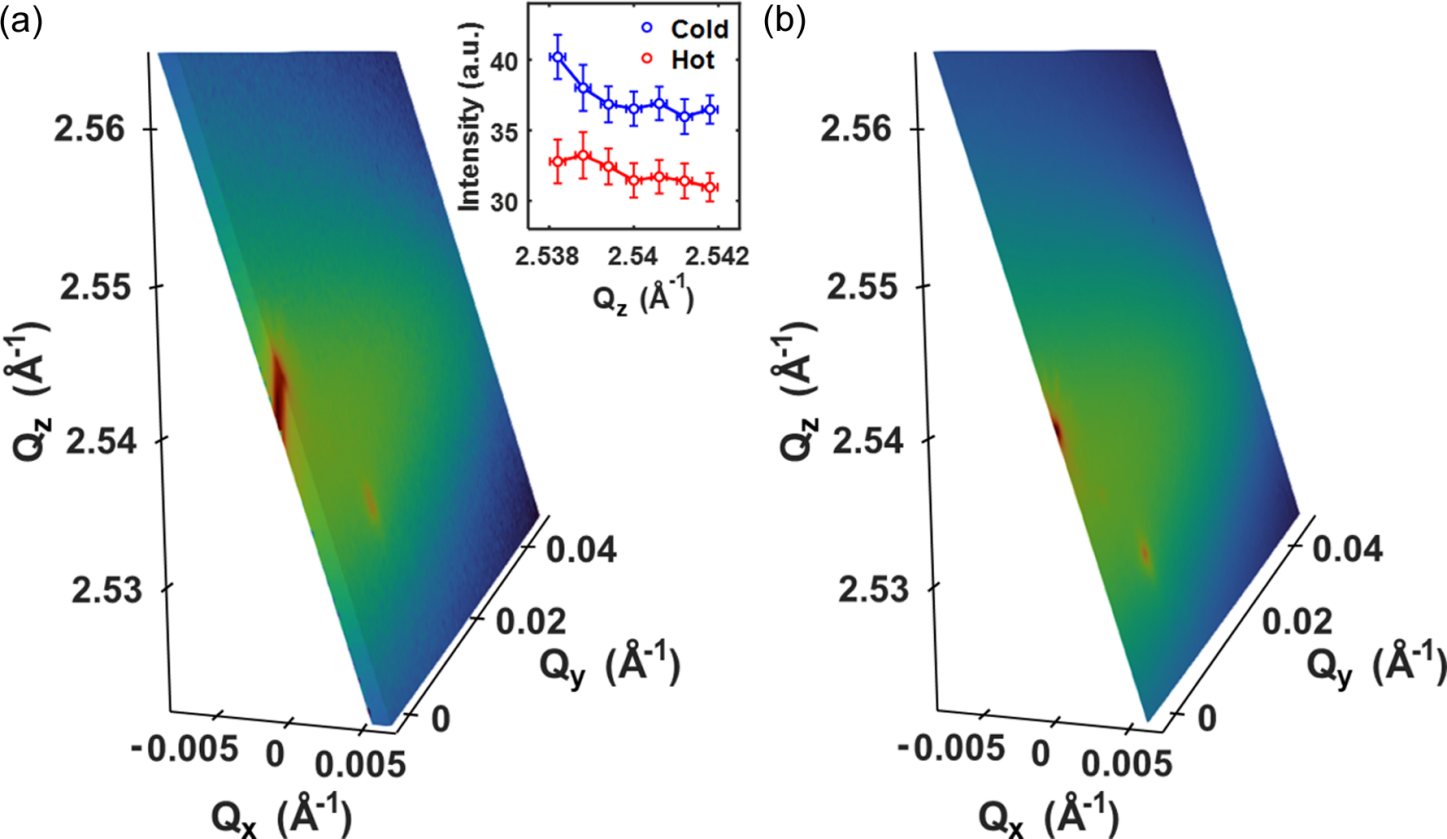
(*a*) Thin shell of the RSM around the NiO(111) Bragg peak captured using the energy-resolved SASE XFEL beam. The probed region includes the intense Bragg rod extended along the film normal direction. The inset compares the Bragg rod profiles in the cold state and excited state by the pump beam at a time delay of 11 ps with the standard deviation. (*b*) 2D RSM around the NiO(111) obtained with the pink XFEL beam without resolving the pulse energy.

Fig. 4 ▸(*a*) illustrates the RSM of the NiO (111) in the cold state reconstructed from the data obtained using 150 pulses whose energy was resolved individually using the proposed procedure. The probed region consists of a thin shell in reciprocal space including the intense Bragg rod from the NiO(111) Bragg peak extended along the film normal direction. The intensity profiles along the Bragg rod in the cold state and in the hot state, 9 ps after pumping, are shown in the inset although limited in range. This suggests that the change of the Bragg rod profile due to pump excitation can be studied together with the change in the diffuse scattering. In comparison, the RSM obtained using the average energy of all pulses without resolving each pulse energy is depicted in Fig. 4 ▸(*b*). We note that the probed region is limited to a 2D sheet in reciprocal space and the Bragg rod is represented only by a single point.

**Figure 5:**
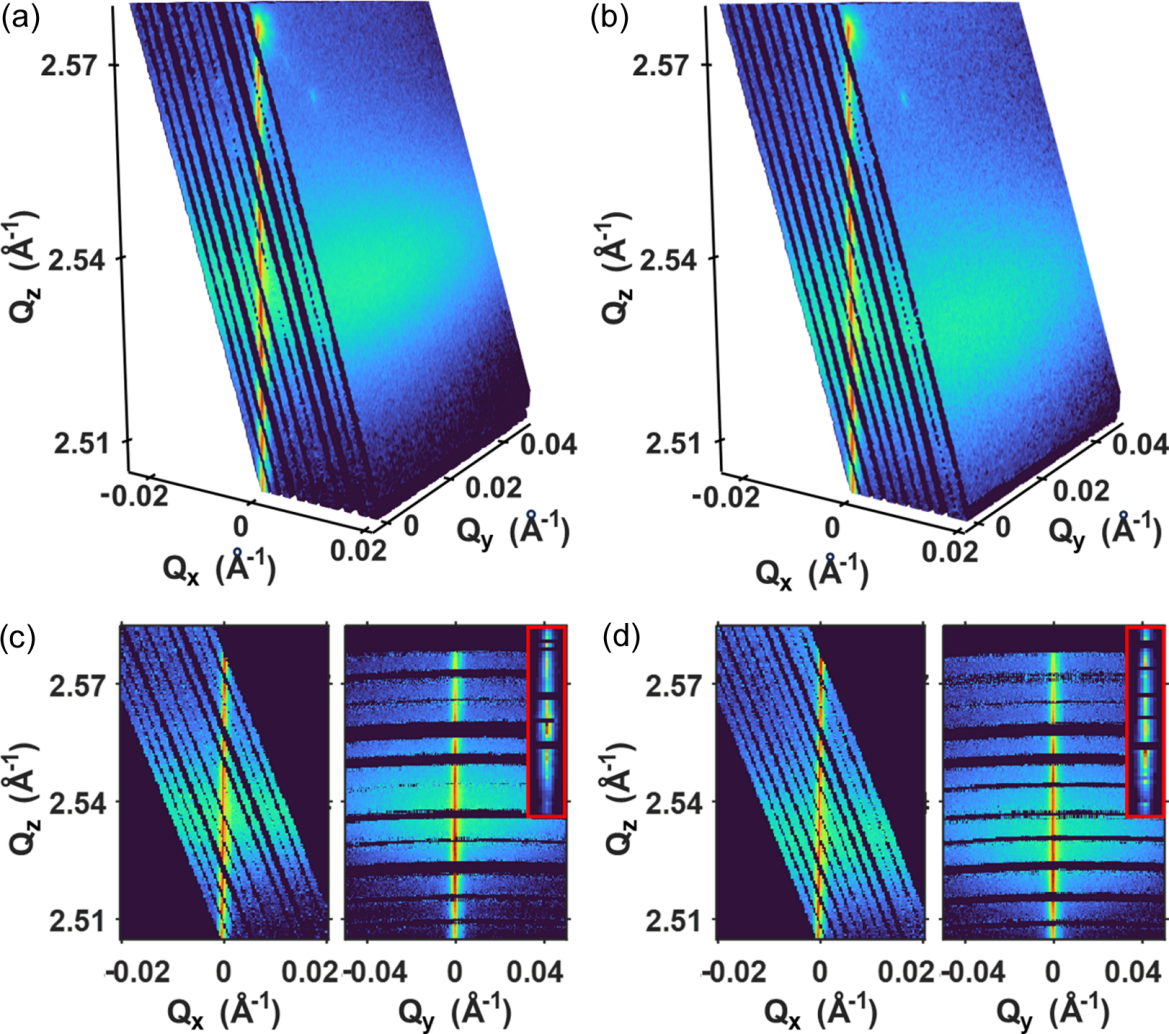
Extended 3D RSM of the (*a*) cold and (*b*) hot states at 9 ps delay obtained using the energy-resolved pink XFEL beam and rocking the specimen. Each slice was obtained at a fixed specimen angle and the color expression is the same as that used in Fig. 4 ▸. Diffraction profiles in the (*c*) *Q*_*x*_–*Q*_*z*_ and (*d*) *Q*_*y*_–*Q*_*z*_ planes obtained from the 3D RSM shown in (*a*) and (*b*), respectively. Insets on the right-hand side panels are magnified images of the Bragg rod, *Q*_*z*_ ranges from 2.505 to 2.555, with a narrower range in the color map. Pixels in insets are vertically binned for visibility

Due to the limited energy band, the reciprocal space probed by the pink XFEL beam is confined to a part of a rather thin shell on the Ewald sphere as shown in Fig. 4 ▸(*a*). The probed range of reciprocal space was 0.004 Å^−1^ in the film normal *Q*_*z*_ direction. This probing volume can be extended by rocking the sample, as in a typical RSM measurement. The extended probing volume is shown in Figs. 5 ▸(*a*) and 5 ▸(*b*), obtained by rocking the NiO sample with 0.05° steps in the ±0.25° range, for both the cold [Fig. 5 ▸(*a*)] and the hot states at a time delay of 9 ps [Fig. 5 ▸(*b*)]. The gaps between the slices arose because the energy band of the pink beam was insufficient to cover the reciprocal space between adjacent rocking angles, and can be eliminated by reducing the rocking angle step. As we obtained the 3D RSM, it is straightforward to investigate the diffraction profile in arbitrary planes and directions. Figs. 5 ▸(*c*) and 5 ▸(*d*) show the diffraction profiles in the *Q*_*x*_–*Q*_*z*_ and *Q*_*y*_–*Q*_*z*_ planes that exhibit the diffuse scattering and the Bragg rod more clearly.

**Figure 6:**
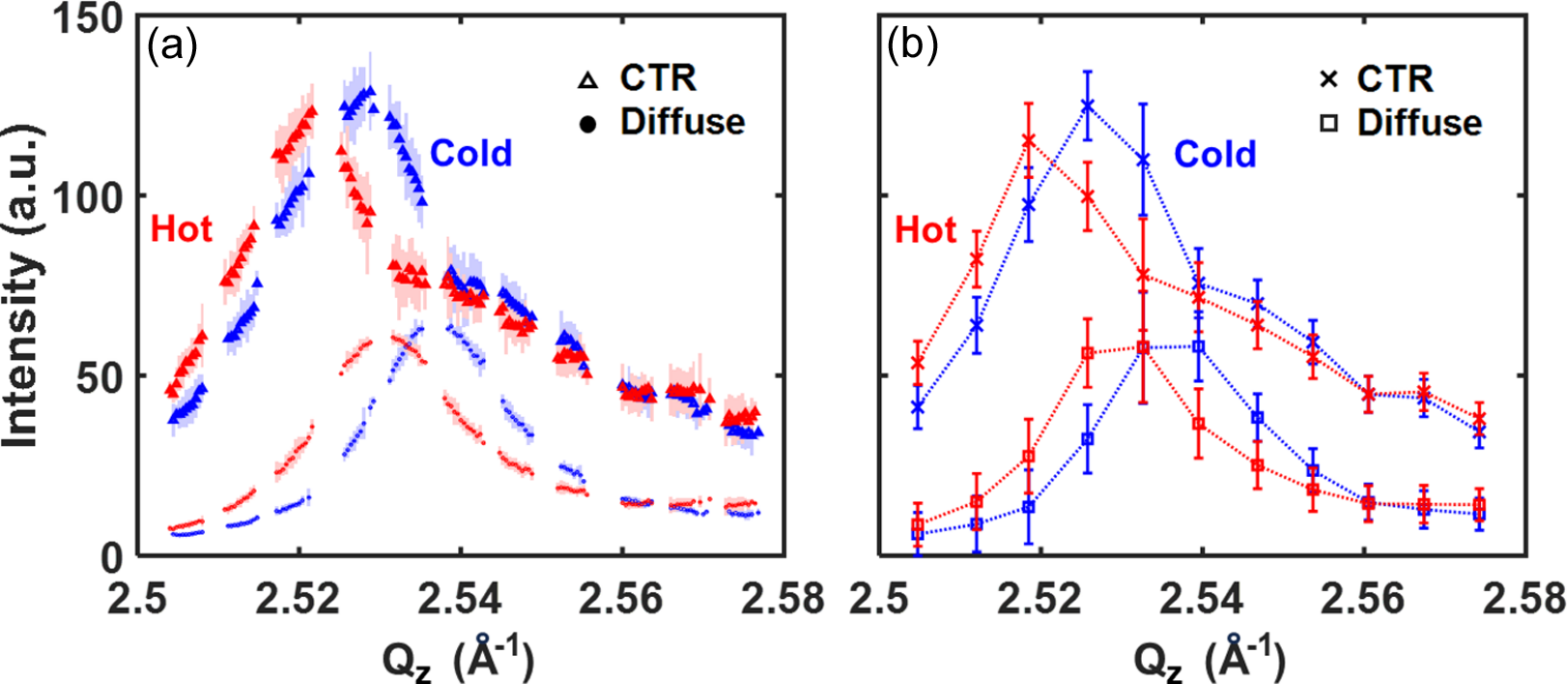
(*a*) Bragg rod and diffuse scattering profiles of the hot (9 ps after pumping) and cold data along the normal *Q*_*z*_ direction obtained with energy-resolved pulses. The 95% confidence interval is indicated by the shaded areas. Diffuse scattering is integrated in 0.003 Å^−1^ × 0.007 Å^−1^ in the *Q*_*x*_–*Q*_*y*_ plane. (*b*) Bragg rod and longitudinal diffuse scattering profile obtained without resolving the pulse energy. The 95% confidence interval is indicated by error bars.

The Bragg rod as well as the longitudinal diffuse scattering along the normal *Q_z_* direction are well resolved as shown in the line profiles exhibited in Fig. 6 ▸(*a*), even with only 11 steps of rocking angle as there are 150 points probed by the pulses with distinct energies at each angle. These points are binned into intervals of 0.0004 Å^−1^ in this plot. This is in contrast to the discrete 11 points obtained without resolving energy in Fig. 6 ▸(*b*), which shows the overall shape but not the details. After pumping, both the Bragg and the diffuse peaks shifted towards the low-*Q* direction, which is related to the propagation of tensile strain on the picosecond timescale. Detailed analysis of the ultrafast time evolution of the NiO film will be reported in the future.

One might think of using a monochromated SASE beam, sacrificing photon flux in return for a more accurate pulse energy, and fill in data between the scarce points in Fig. 6 ▸(*b*) by increasing the number of steps in specimen rocking. However, it is generally not an option in typical pump–probe experiments as one needs to repeat the rocking scan at each time delay, which is very time-consuming. If we use a conventional scenario using a monochromatic beam to obtain the data in Fig. 6 ▸(*a*) in the same RSM range covered by the proposed method at a single rocking angle, approximately seven steps of specimen rocking would be required. Furthermore, to achieve comparable diffraction intensity with a monochromated SASE beam especially for weak diffuse scattering, a significant number of shots should be summed. Given that the photon flux of the monochromatic beam is about 30 times smaller than that of the pink beam, roughly 4500 exposures to the pump laser and XFEL beam would be necessary to achieve the same intensity level as the data shown in Fig. 6 ▸(*a*), obtained using only 150 exposures. The measurement time would increase proportionally to the number of shots, added by the time required for diffractometer motion. With the progress of seeded XFELs of high spectral brightness, the limitation due to the weak scattering can be overcome to some degree. Nonetheless, increasing exposure to intense pumping laser pulses in order to use a monochromatic beam and rocking specimens may cause further damage to the specimens (Lee *et al.*, 2019 ▸; Stuart *et al.*, 1996 ▸), which hinders the integrity of data obtained in a pump–probe fashion.

Considering the advantages of the proposed 3D RSM using the SASE beam, the method would be impactful in studying systems whose thorough dynamic can only be observed in pump–probe 3D RSM methods. For example, ultrafast strain dynamics in thin films (Zeuschner *et al.*, 2024 ▸; Gu *et al.*, 2023 ▸), dynamics involved in structural phase transitions where one needs to follow the detailed change of a Bragg peak and rod, could be investigated without scanning the diffraction angle. Studies with specimens vulnerable to pump laser beam irradiation will be especially benefited by this method.

Since this methodology relies on the features in the diffuse scattering that arise from mosaicity and finite structural correlations within a specimen, it would be challenging to apply it to structurally perfect specimens, such as single crystals or super-thick structurally coherent films. Additionally, for specimens with very sharp diffuse features, the energy coverage range may be smaller than the inherent pink beam energy bandwidth. For highly strained samples, it is essential to determine their lattice spacing prior to pump–probe measurements and ensure that the strain in the cold state remains unchanged.

## 4. Conclusions

We demonstrated a pump–probe methodology utilizing XFEL pink beams in order to achieve 3D RSM by resolving the energy of individual pulses. Based on the fact that odd- and even-numbered pulses share the same average energies for a sufficient number of pulses, we were able to resolve the energy of individual pulses using the specimen’s diffraction pattern as a spectroscopic reference during pump–probe measurements. This methodology was demonstrated at the PAL-XFEL with NiO thin film specimens, observing the NiO (111) Bragg peak. A thin slice of reciprocal space within the pink beam energy bandwidth was successfully probed by the energy-resolved pulses. Additionally, more extensive 3D RSMs were obtained by rocking the specimen. As a result, it was possible to follow the time evolution of the NiO (111) peak in 3D reciprocal space and its attributes were extracted, including changes in the Bragg rod and diffuse scattering which would not have been obtained without resolving the energy of each pulse.

The 3D RSM method using the SASE beam presented in this paper would be beneficial in investigating various ultrafast structural dynamics of practical materials. X-ray absorption near edge spectroscopy may be combined with the proposed method to investigate the ultrafast edge shifts of oxide materials during oxidation or reduction, which are typically on the order of a few electronvolts. However, careful analysis is required due to the limited energy bandwidth and resolution.
